# Understanding patient participation behaviour in studies of COPD support programmes such as pulmonary rehabilitation and self-management: a qualitative synthesis with application of theory

**DOI:** 10.1038/npjpcrm.2015.54

**Published:** 2015-09-17

**Authors:** Ratna Sohanpal, Liz Steed, Thomas Mars, Stephanie J C Taylor

**Affiliations:** 1 Centre for Primary Care and Public Health, Blizard Institute, Barts and the London School of Medicine and Dentistry, Queen Mary University of London, London, UK

## Abstract

**Background:**

In chronic obstructive pulmonary disease (COPD), the problem of poor patient participation in studies of self-management (SM) and pulmonary rehabilitation (PR) programmes (together referred to as COPD support programmes) is established. Understanding this problem beyond the previously reported socio-demographics and clinical factors is critical.

**Aims:**

The aim of this study was to explore factors that explain patient participation in studies of COPD support programmes.

**Methods:**

Thematic ‘framework’ synthesis was conducted on literature published from 1984 to 1 February 2015. Emergent themes and subthemes were mapped onto the adapted ‘attitude–social influence–external barriers’ and the ‘self-regulation’ models to produce analytical themes.

**Results:**

Ten out of 12 studies were included: PR (*n*=9) and SM (*n*=1). Three descriptive themes with 38 subthemes were mapped onto the models' constructs, and it generated four analytical themes: ‘attitude’, ‘social influences’ and ‘illness’ and ‘intervention representations’. The following factors influenced (1) attendance—helping oneself through health improvements, perceived control of worsening condition, perceived benefits and positive past experience of the programme, as well as perceived positive influence of professionals; (2) non-attendance—perceived negative effects and negative past experience of the programme, perceived physical/practical concerns related to attendance, perceived severity of condition/symptoms and perceived negative influence of professionals/friends; (3) dropout—no health improvements perceived after attending a few sessions of the programme, perceived severity of the condition and perceived physical/practical concerns related to attendance.

**Conclusions:**

Psychosocial factors including perceived practical/physical concerns related to attendance influenced patients’ participation in COPD support programmes. Addressing the negative beliefs/perceptions via behaviour change interventions may help improve participation in COPD support programmes and, ultimately, patient outcomes.

## Introduction

Programmes such as pulmonary rehabilitation (PR) and self-management (SM) education programmes that support patients with chronic obstructive pulmonary disease (COPD), to increase their skills and confidence to better self-manage their condition, are established treatments alongside pharmacological treatment.^1–4^ However, studies of these programmes report poor patient participation and high dropout rates,^5–7^ with similar problems acknowledged in clinical practice.^8^ Understanding the problem of poor patient participation and retention is therefore critical^9^ before strategies to improve participation can be suggested.

Our recent review^10^ highlighted that ‘participation’ is a term used differently by different researchers. Here, by ‘participation’ we mean ‘taking part in a study or intervention’ with ‘attendance’, ‘non-attendance’ and ‘dropout’, all aspects of participation—where attendance means ‘exposed to at least part of the intervention’; non-attendance means ‘exposed to no part of the intervention’; dropout means ‘dropped out from the intervention’; completion means ‘completing the study or intervention’.

A mixed-methods review^11^ of participation in PR programmes attributed patient non-attendance and dropout to personal, clinical, social and physical barriers. Only one study^12^ of a COPD SM programme has explored reasons for high or low attendance, and the findings comprised a mix of socio-demographic, personal and clinical factors. It has been suggested that socio-demographic and clinical factors may be insufficient to understand the problem of poor participation in these programmes; a new approach is therefore needed.^6,8,13^

We propose that an approach that views participation as a health behaviour and that uses health behaviour theory and constructs related to behaviour change could further our understanding of participation behaviour.^14^ Such an approach could help identify appropriate targets for an intervention,^13^ with the ultimate aim of improving patient participation in PR and COPD SM programmes, thus enhancing patient outcomes.

Health behaviour theory has been used in several studies^15–23^ to explain or predict participation, particularly attendance in patients with a variety of conditions; however, only one study^20^ has used such an approach in COPD.

The aim of this qualitative synthesis was to explore the factors that might explain patient participation in studies of COPD SM and PR programmes. PR, including provision of individually prescribed lower limb exercise training,^24^ encourages and assists patient SM by promoting decision-making and self-efficacy through SM education in goal setting and setting an action plan.^3^ SM education is integral to the delivery of SM programmes.^7^ Hence, these will be together referred to here as COPD support programmes. The research questions were as follows:

1. What are the possible factors affecting patient participation in COPD support programmes?
2. Can behavioural theory help explain patient participation in COPD support programmes?

## Materials and methods

The qualitative synthesis sat within a broader review of participation,^10^ which followed recommended guidance for conducting systematic reviews;^25^ one search strategy was applied to identify both qualitative and quantitative studies (Supplementary Appendix 1). All qualitative research studies from 1984 to 1 February 2015, exploring reasons for participation in studies of PR, SM and health education programmes, including in the programme, by patients with COPD were considered for inclusion. We included studies that explored patient reasons for not attending their initial PR assessment and patient views of participation before the start of the programme.

Data extraction (Supplementary Appendix 1) included findings of each primary study.^26^ We used the modified critical appraisal skills programme checklist^27^ for quality assessment.

Thematic ‘framework’ synthesis, an established method, was conducted.^26^ The synthesis involved three distinct stages.^26,28,29^

**Figure 1:**
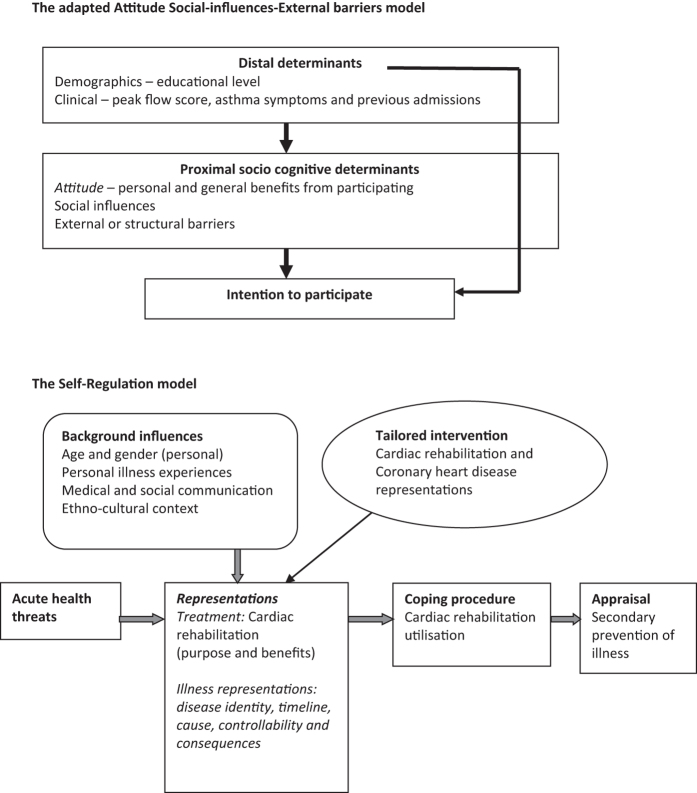
The ‘best fit’ theoretical models. **The adapted ‘attitude–social influence–external barriers' model.** Lemaigre *et al.*^16^ reported using the ‘attitude–social influence–self-efficacy’ model by de Vries *et al.* to explain intention to participate in an asthma self-management programme. The constructs as measured by Lemaigre were as follows: attitude including personal and general benefits of the asthma programme. Social influence including social norms to take care of their asthma and motivation to comply with these. Self-efficacy (external barriers) including beliefs about barriers to participate. It should be noted that the definitions of these constructs vary slightly from those used in the original de Vries model (see Supplementary Appendix 2 for further details), particularly that of self-efficacy, which focuses primarily on external barriers and hence is labelled as such here. The figure above illustrates the distal, proximal socio-cognitive and external constructs of the adapted model that explained intention to participate in an asthma self-management programme in the study by Lemaigre *et al*.^16^ The descriptive themes and subthemes were ‘mapped’ onto the adapted model’s theoretical constructs. The ‘self-regulation’ model. Keib *et al.*^21^ used the ‘self-regulation model’ by Leventhal *et al.* and the ‘necessity-concerns framework’ by Horne to explain participation in cardiac rehabilitation among patients with coronary heart disease. **The self-regulation model** explains the effort an individual makes, in response to a health threat, to protect and maintain health and to avoid and control illness based on representations of the illness. The threats (physical and psychological indicators) that disrupt physical and cognitive function as a result of the illness make contributions to the illness representation. Leventhal *et al.* in the 1990s stated the five domains of ‘illness representations’: 'disease identity' is the perceived symptom experienced as a result of an illness and the symptoms associated with the illness. *'*Timeline' is the perceived expected duration of illness (acute and chronic) or expected age of onset of illness. 'Consequences' is the perceived severity and impact on life functions as a result of the illness. Cause could be perceived as internal (e.g., genes) or external (e.g., infection). Control/cure is whether the illness is perceived as ‘preventable’, ‘curable’ or ‘controllable’. These illness representations can lead an individual to generate goals, and to develop and carry out action plans (referred to as coping procedures), which are subsequently evaluated in relation to whether the threat has been eliminated or controlled and influence subsequent representations of the illness and behaviour and hence self-regulation. Horne within the ‘necessity-concerns framework’ stated that 'necessity' beliefs are perceived personal needs for the treatment and 'concerns' beliefs are perceived concerns of the treatment, both of which influence treatment adherence. The figure above illustrates the illness and treatment representations that explained participation in cardiac rehabilitation in the study by Keib *et al.*^21^ The descriptive themes and subthemes were ‘mapped’ onto the above-mentioned theoretical constructs. Supplementary Appendix 1 describes how Keib used the model to explain participation.

1. The findings of each included study were coded to generate free concepts with review questions in mind. The emergent concepts from one study were compared with those from other included studies, which was referred to as the translation of concepts.
2. The emergent concepts were examined for similarities and differences, and then grouped and placed under new codes that captured the meaning of the grouped concepts to produce ‘descriptive themes’ and subthemes.
3. These descriptive themes and subthemes were ‘mapped’ onto two *a priori* theoretical models with subsequent ‘generation of analytical themes’ that went beyond the findings of the original studies. An *a priori* ‘framework’, characteristic to framework synthesis, is informed from the literature and team discussion to synthesise findings.^28^ We applied the recommended ‘best fit’ framework synthesis approach,^29,30^ whereby an existing conceptual model, which most closely matches the research topic under study, is used to carry out the synthesis. Using the ‘best fit’ approach helped us in limiting our selection among the numerous and varied behaviour change theories to identifying two most applicable health behaviour theories, as they had previously been used in studies to explain participation behaviour of patients with chronic disease in SM support programmes. We used the adapted ‘attitude–social influence–external barriers’ model that has previously been used to explain intention to participate in an asthma SM programme^16^ and the ‘self-regulation’ model that has been used to explain cardiac rehabilitation utilisation^21^ and examined whether our results were consistent with either or both these models. Figure 1 briefly describes these two models and Supplementary Appendix 2 elaborates on the models.

## Results

**Figure 2:**
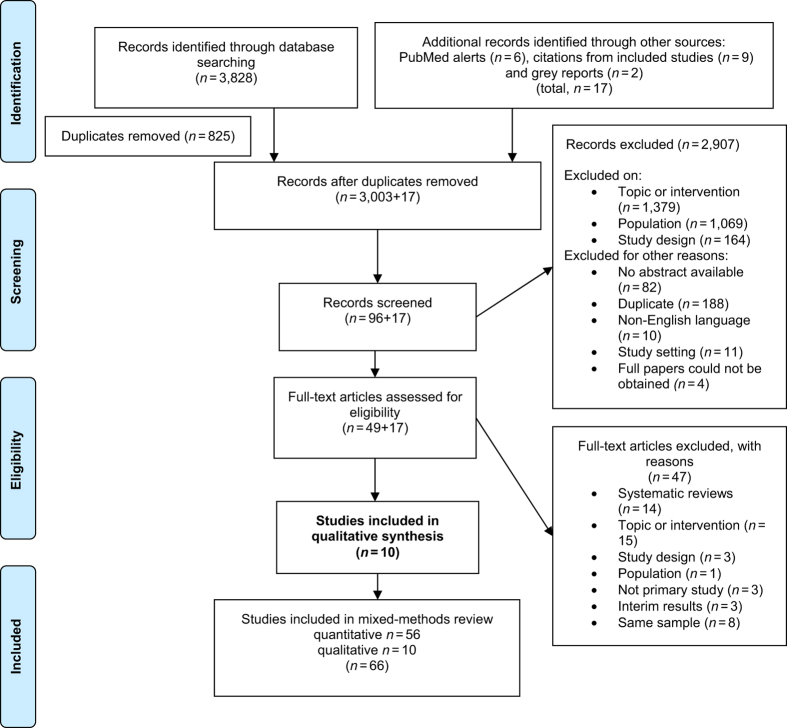
PRISMA flowchart.^31^

Ten studies were included in this review (Figure 2).^31^ Two studies^32,33^ were excluded because patients’ reasons for participation were not explored. Nine of the included studies examined PR programmes,^11,34–41^ and only one study examined a COPD SM programme.^12^ Table 1 presents the study characteristics.

### Quality appraisal

The agreement between the authors on the modified critical appraisal skills programme checklist score was 100%. There was variation in study reporting—e.g., lack of clear reporting on sampling strategy,^34–36^ the data analysis process,^37^ the research questions^36^ and in some cases the authors’ interpretation of data was not supported with verbatim data.^35,36,38,40^ The level of agreement, for mapping of subthemes onto *a priori* theory, was higher for the adapted ‘attitude–social influence–external barriers’ model (97%) compared with the ‘self-regulation’ model (88%).

### Synthesis findings

The synthesis generated three descriptive themes (with 38 subthemes (Table 2)) related to reasons or potential reasons for:

1. Attending SM and PR programmes and reasons for continuing and completing PR,
2. Not attending SM and PR programmes and
3. Dropping out of SM and PR programmes.

Twenty-nine subthemes were mapped onto both theoretical models; four subthemes were not mapped onto the adapted model and five were not mapped to the ‘self-regulation’ model either owing to limited primary data or lack of correspondence to model constructs. In some cases, subthemes were mapped onto more than one theoretical construct within the same model (Table 3). In addition, overlap between different model constructs was observed (Table 3); for instance, the same subthemes that were mapped onto the ‘attitude’ construct of the adapted ‘attitude–social influence–external barriers’ model were also mapped onto the ‘intervention representations’ construct of the ‘self-regulation’ model.

**Figure 3:**
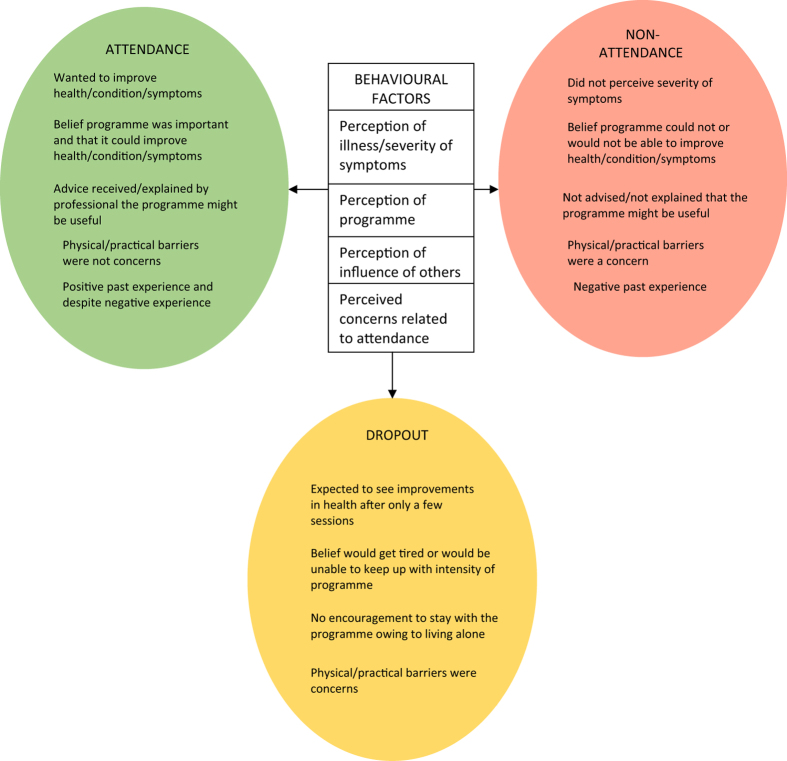
Illustration of factors affecting patient participation in COPD support programmes.

The mapping revealed four key behavioural constructs that formed our analytical themes and explained the three descriptive themes of participation (attendance, non-attendance and dropout) in COPD SM support. In the adapted ‘attitude–social influence–external barriers’ model, patient socio-demographics or perception of clinical symptoms contributed to the formation of beliefs about participation in COPD support programmes, and these beliefs were captured by the ‘attitude’ and ‘social influences’ construct of the model. While in the ‘self-regulation’ model, the perceived health threat (COPD) along with the personal illness experience and the medical and social communication led to the formation of perceptions about the illness and the intervention (COPD support programmes) which led to participation; these perceptions were captured by the ‘illness representations’ and ‘intervention representations’ constructs of the model. These findings went beyond the findings of the included primary studies. The analytical themes are described below. Figure 3 illustrates the behavioural factors that can affect participation behaviour.

### Attitude

The attitude of attenders was that COPD support programmes^12,34–36^ could help improve their health and condition. Many participants wanted to help themselves^34,41^ and wanted to learn about their condition.^12^ Some participants wanted to gain control of their condition.^34–36,39^ A few wanted to cope with the illness and remain independent.^36,39^ Besides perceived health benefits, social benefits were important too.^12,34^ Some participants saw COPD support programmes^12,34^ as a reason to get out of the house, to socialise and meet others with the same illness. Two interviewees reported attending the SM programme for altruistic reasons.^12^

In the studies by Arnold *et al.*^34^ and Guo and Bruce,^41^ the findings were sub-divided into reasons for ‘attending’ or ‘continuing and completing’ PR (Table 2); a key reason given for continuing and completing PR was social benefits and health benefits including those that were seen immediately following application of skills learnt in PR. Only *Fischer et al.*^35^ compared reasons for attending PR in brand new referrals and those who had previously attended the programme. Among the previous attendees, positive past experience of PR, particularly staff supervision and support, influenced attendance. A few patients in the study by Bulley^39^ had a negative PR experience; however, this did not stop them from having a positive attitude to attending PR again.

In contrast, the attitude among non-attenders was that PR was not beneficial, particularly that the exercise component would not improve health or could not improve health.^11,38,40^ Several participants in Taylor’s study^38^ were more interested in research testing new drug treatments and not exercise. Some participants in the same study chose not to attend PR because they perceived the exercise negatively, such as too vigorous, strenuous and detrimental to their health. The majority of participants in the study by Guo^41^ believed that exercise would make them become breathless. Furthermore, among some interviewees, personal negative experience with exercise in the past such as embarrassment, panic, lack of control and negative research experience led to negative attitudes and were other reasons suggested for PR non-attendance.^38,39^

With regard to dropout, some participants^11,37^ dropped out because they did not perceive any health improvements after attending between one and four PR sessions, and therefore they had the attitude that PR was not beneficial. Failing to notice any improvements in health halfway through the programme was also suggested as a potential dropout reason in the study by *Fischer et al.*^35^ (Table 2). Other potential dropout reasons suggested in the study by *Fischer et al.*^35^ were as follows: inability to keep up with the intensity of the programme and feeling uncomfortable while training with other participants. In two other studies^11,40^ a few participants dropped out because they were too tired to complete the programme or did not believe that they could continue with the exercises despite being told what to expect.

### Social influences

Non-attendance in PR was influenced by a lack of positive feedback or a lack of explanation given on the benefits of the programme. Several participants decided not to attend PR because their friends or family either had not found or they did not think PR would be useful.^38^ Another trusted source, health professionals, being unable to explain or advise participants about the benefits of PR or not giving any information on what the programme might entail was associated with non-attendance.^11,34,35,40^

Conversely, the majority of attenders^34,35,37^ attended PR because their doctor was enthusiastic about the referral and thus they believed that the programme would be useful, they were explained how the programme could benefit them or they simply trusted the advice or suggestion to attend the programme. A referral to PR was enough for some participants to attend PR.^39,40^

### Intervention representations

A positive perception/representation of COPD support programmes influenced attendance. Some participants perceived that the SM programme would help them learn about SM.^12^ PR was perceived as a positive step to help oneself; participants believed that attending PR would help them gain control of their condition.^34–36,39^ A few participants saw PR as their only hope of coping with the disease and remaining independent.^36,39^ Perceived benefits from PR attendance in the past also influenced attendance,^35^ and for a few participants the negative experience did not deter them from wanting to attend PR again.^39^ In addition, almost all attenders in the study by *Fischer et al.*^35^ perceived PR as a necessity if they wanted to see improvements in their health and were not concerned about their conflicting obligations.

In contrast, the perceived negative effects of exercise/PR^11,38,40,41^ and previous negative experience with exercise in the past influenced non-attendance among several participants.^38,39^ Non-attendance in COPD support programmes was also influenced by participants’ perceived environmental concerns related to attendance, such as a complex journey that involved using more than one bus to get to the venue,^40^ unable to access a car or public transport^11^ or difficulty in using public transport or parking, particularly for people with restricted mobility, who were housebound/wheelchair bound or relied on gait aids;^11,12,35,37,38^ location (hospital) of the programme;^38,40,41^ seasonal weather;^38^ practical issues such as personal/professional commitments.^11,12,35,37,38,40^ A few participants indicated that the PR was held too early in the day, and this also affected attendance.^11^

Some patients dropped out of PR because they expected to see health improvements after only a few sessions.^11,37^ Some patients dropped out because they felt too tired to complete the programme; a few patients perceived that they would get tired^11^ or they would be unable to continue with the exercises despite being told what to expect.^40^ Similar to non-attenders, dropouts were also concerned about issues of access,^11,35^ including the cost of relying on taxis or of parking, the programme being held too early in the day and competing demands.^11,40^ In addition, not perceiving any benefits when one was halfway through the programme, an intensive programme and being uncomfortable while training with others in the group were cited as potential dropout reasons.^35^

### Illness representations

The perceived increased severity of condition and its perceived consequences such as effect on ability to cope/self-manage, partake in social activities, be in control and remain independent prompted attendance by several participants in COPD support programmes.^12,34–36,40,41^

Among non-attenders, some participants felt that they were too disabled to carry out any sort of activity either because of COPD or other co-morbidities^37,39,40^ or to leave the house without support;^12^ some felt that their health needed to improve to enable them to attend;^11,41^ some perceived that improvements in their health were no longer possible;^38,41^ some feared that their existing condition/s was getting worse.^11,35^ Conversely, some participants did not perceive their health or condition to be poor or serious enough to warrant attendance.^34,38,40^

The perceived severity of symptoms also influenced patient dropout behaviour. Suffering an acute exacerbation of COPD or other conditions often led participants to drop out of PR, as they needed time to recover.^11,37,41^ A couple of participants dropped out of the SM programme because of depression associated with their condition.^12^ Conversely, a couple of participants in the latter study dropped out because they did not perceive themselves to be physically or psychologically affected by their condition.

## Discussion

### Main findings

The thematic ‘framework’ synthesis with the use of health behaviour theory helped in gaining an insight into the participation (attendance, non-attendance and dropout) behaviour of patients in COPD support programmes beyond the previously reported socio-demographic and clinical factors. The mapped subthemes yielded higher-order constructs, whereby participation was influenced by an individual’s attitude and perceived social influences, as well as intervention and illness representations.

Attitudes of wanting to help themselves, the perceived influence of health professionals that the programme might bring health improvements, perceptions of the controllability of illness and gaining independence and perceived positive benefits of the COPD support programmes, including past experiences, influenced attendance behaviour. Non-attendance was influenced by a negative attitude that health improvements were no longer or could not be possible, negative perceptions that exercise would not benefit the condition, and past experiences such as perceived physical/practical concerns related to attendance, a lack of information about the programme from professionals, and the negativity of professionals and family/friends towards the programme. Dropout behaviour was influenced by unmet expectations after attending only a few sessions of the programme, perceived severity of symptoms and perceived physical/practical concerns related to attendance.

### Interpretation of findings in relation to previously published work

In agreement with studies that have utilised the ‘self-regulation model’ to predict or explain patient participation in rehabilitation among patients with chronic disease including COPD,^15,18,20,21^ attendance was associated with patients’ perceptions in self to control, gain independence and cope with their condition, and patients' perception of the COPD support programme was perceived as necessary to help control and improve their condition. The same was explained by patients having a positive ‘attitude’ towards the programme in this review and among other studies that have used the adapted ‘attitude–social influence–external barriers’ model^16^ and the theory of planned behaviour^42^ to explain patient participation in the asthma SM programme^16^ and cardiac rehabilitation.^42^ However, the lack of perceived benefits from attending PR was reported among non-attenders in this study, and it has been reported elsewhere for patients with chronic disease in rehabilitation programmes.^15,21,42,43^

In addition, we found that a positive past experience with exercise influenced attendance and completion of PR and negative experiences with exercise in the past influenced non-attendance. Within a behavioural context, the benefits gained from previous experience may have led to the formation of positive beliefs about PR, and these beliefs contributed to the appraisal of ‘attending’ PR as positive. The positive appraisal was retrieved^44^ after invitation to attend PR, which might have led to attendance. Conversely, the reverse could have resulted in non-attendance. However, for two participants in this study a past negative experience of PR did not prevent them from wanting to attend PR again. This has been explained by the participants having a positive attitude towards the programme and a result of having positive interactions with health-care professionals.^39^

A referral to PR, and in addition referral from enthusiastic health-care professionals who gave advice, suggestions or explanations on how the programme might benefit health or their condition, influenced many patients to attend PR in this review. In contrast, patients who did not remember the referral and professionals who provided no explanation on how the programme could help the patient or used words such as ‘intensive’ or ‘there’s nothing we can do for you’, or family and friends who associated the programme as a ‘waste of time’, were negative influences, and this led to patient non-attendance. Patients with COPD have faith in their health professionals,^39^ and studies^16,42^ have shown that rehabilitation attenders strongly believed that their doctor, family or friend wanted them to attend rehabilitation. This suggests that to enable programme attendance, professionals, family and friends should work together or initiate a ‘dialogue’ with a patient, identify their needs^45^ and accordingly advise on programme benefits that other patients might have experienced, such as social and psychological benefits,^43^ improvements in activities of daily living,^46^ self-esteem and self-worth,^46^ and strength, balance and flexibility.^47^ Recently, advice or individual counselling from trained health-care professionals in primary care settings that include behaviour change strategies, such as establishing objectives and writing physical activity prescriptions, have been reported as most effective in promoting, changing or increasing health behaviours.^48,49^ Perhaps these strategies could also be applied to improve attendance behaviour in COPD support programmes. Advice about PR benefits through peer support has also been recommended in studies to increase programme attendance.^11,40^ This latter form of support may particularly help improve PR adherence among people who live alone and require encouragement and confidence to continue their attendance, which was found in this study.

Non-attendance and non-completion has previously been explained in terms of the value ascribed to PR, minimal value or low relative value in view of other more important values, burdens and costs are reported among non-attenders and high relative value among non-completers who may be more likely to consider attending PR if other values or burdens could be addressed.^11^ Behaviourally, we were able to explain this another way, whereby some attenders perceived COPD support programmes as necessary to attend, as they were clear on its benefit and were not concerned about the practical/physical barriers related to attendance. In contrast, some non-attenders were unclear or did not believe in the benefits of exercise for their health, and non-completers did not perceive health improvements after attending one to four sessions (of a twice weekly, 6–12-week programme),^24^ and both non-attenders and non-completers perceived several practical/physical concerns related to attendance. By assessing, eliciting and understanding patient perceptions about COPD support programmes, we might be able to identify patients who could be targeted by behaviour change interventions aimed to improve programme uptake^19^ such as changing or challenging patients’ beliefs and misconceptions^3,50^ before programme attendance^3^ and having physical/practical resources^50^ in place to enable participation—e.g., programme commissioners could plan for payment of patient transport, thereby helping to reduce the burden of travel and costs from the patient.^24^ Home-based services or PR have been suggested by studies^11,40^ based on patient preference for PR completion; however, this alternative should not be aimed at patients with low motivation or low interest,^51^ which could be determined during patient assessment.

Some patients in this review dropped out of PR after a few sessions because of unmet expectations in terms of health improvements, which could be because they might not have been told what to expect from PR;^52^ however, the intensity of the programme was another reason that led some patients to drop out despite them being told what to expect from the programme before attendance. This suggests that perhaps the PR staff could follow-up patients after the end of the first few sessions to identify any patient issues or concerns related to exercise/programme and attendance.^19^ Here again negative beliefs might need targeting using behaviour change interventions^19,50^ or more resources might be needed—e.g., more staff for supervision of patients who might be more disabled or in need of more attention.^24^ The support offered by PR or structured exercise programme staff has been deemed important for completion of the programme by patients with chronic disease^43^ and older people.^47^ A recent study^53^ to maintain adherence in a hospital-based smoking cessation service evaluated a booklet that presented information about the effectiveness of the service (presented smoking cessation rates of the service alongside those achieved nationwide) and asked participants to read and tick pre-defined implementation intentions (or ‘if-then’ plans) and found that the booklet improved adherence but not the intention implementation plans. The authors attributed this to patients’ high socio-economic status and reading and ticking pre-designed ‘if-then’ statements rather than forming individualised plans that are associated with behaviour change. COPD is a disease mostly associated with people belonging to low socio-economic status^54^ and they are widely reported to have poor literacy and health literacy.^55^ As a result, trained staff working with patients in partnership to develop understanding of benefits and develop tailored ‘if-then’ implementation plans before programme attendance may help to improve participation in COPD support programmes.

Finally, we found that while perceived severity of the condition or symptoms influenced patient attendance in COPD support programmes also reported elsewhere,^18^ the perceived severity of symptoms and lack of the fear of symptoms getting worse or not yet having recovered mentally or physically owing to previous exacerbation of COPD or other conditions led to patient non-attendance and dropout in this study. The perception of less severity of symptoms has been reported previously among non-attenders in cardiac rehabilitation.^56^ Patient participation might improve if patients who perceive severity of symptoms can be reassured using either a theoretically worded letter^57^ or explained in person about the aim of COPD support programmes, the immediate benefits that they might be able to see^41^ or shown by invitation to attend a trial session.^58^ In addition, patients should be given enough time and space to recover from their exacerbation, and readiness to attend should be assessed and addressed followed by appropriate referrals to help improve patient participation.

### Strengths and limitations of this study

A strength of this study is that it is the first qualitative systematic review to include 10 qualitative primary studies that aimed to understand patients’ views on each aspect of participation behaviour in COPD support programmes. Previously, a mixed-methods review that explored PR non-attendance and non-completion had included five studies,^5^ three of which were included in this study. However, because of limited resources, only papers in the English language were included in the review. Only one study of a COPD SM programme was included in the review; hence, the findings unique to PR and SM programmes have been specified throughout to make it clear to readers. The included studies were not underpinned by the health behaviour theory; however, this qualitative synthesis with application of two theories to the studies’ findings has made a contribution towards understanding the cognitions that may influence attendance, non-attendance and non-completion behaviour, and has helped to identify key behavioural constructs that can be targeted to improve patient participation.

Some aspects of method reporting were insufficient in some studies—e.g., limited verbatim data. This could affect the transferability of the findings in practice.^59^ However, the study participants were the right people to answer the research questions,^60^ and in line with the review aim we were able to identify a breadth of reasons given for each aspect of participation in COPD support programmes. The inadequate data also prevented mapping of a few subthemes onto the behavioural models constructs.

Both the ‘best fit’ theoretical models were able to explain patient participation to a considerable extent, and our review findings were consistent with both these models. However, at times it was challenging to map an individual’s view or beliefs into the distinct theoretical cognitive constructs; e.g., a reason given for participation was mapped onto more than one construct of the same framework. This suggests that an individual’s cognitions are interlinked and help inform an individual’s decision-making to perform certain behaviour.^61^ In addition, using the ‘best fit’ approach described by Carroll *et al.*^29^ limited us to some extent, whereby we used theories previously utilised in studies and not the newer/latest model versions.^62,63^

### Implications for future research, policy and practice

The study findings have implications for improving participation in COPD support programmes including other interventions aimed at chronic disease patients.

In practice, the findings help in understanding that patient beliefs or perceptions of their illness, the COPD support programmes including the physical and practical concerns related to patient attendance, and social influences, can lead to programme attendance, non-attendance, or dropout behaviour. These findings could also be applicable to other interventions such as cardiac rehabilitation, SM training, physical activity/exercise, smoking cessation that also experience poor attendance and completion by patients with chronic disease other than COPD.^18,64,65^ For professionals involved in caring for patients with chronic disease, it highlights the importance of patient engagement^66^ and prioritising discussion about their illness and its treatment^67^ for improving motivation and longer-term participation in the treatment.^19^ During patient engagement, it would be important for health-care professionals to explain the benefits of the programme in relation to the outcome/s patients would like to achieve for themselves, including the benefits that could be expected straightaway after attending a few sessions and in the longer term. Provision of encouragement and reassurance to patients that the programme can help them learn strategies to gain control, cope and remain independent is critical alongside organisation of smoother referrals^68^ and travel arrangements for improvement in patient participation. To help facilitate this, professionals will require provision of training and support,^69^ increasing availability of programmes in areas local to patients and creating awareness and better communication about service provision.^70^

Assessment of patient perceptions during routine consultations has been suggested for COPD.^71,72^ We propose adaptation of the illness perception^63^ and intervention perception questionnaire^19^ commonly used in studies to predict attendance in cardiac rehabilitation. Assessment of patient perceptions will help identify eligible and suitable patients for the treatment and predict attendance in the treatment.^19^ In addition, the negative perceptions towards illness and treatment^73^ could be targeted using effective behaviour change interventions^48,53,57^ to help improve participation in COPD support programmes. To get health professionals and indeed the wider health system to ‘buy-in’ this form of patient support, exploring the views of professionals (beyond factors affecting patient referral or perceived patient challenges in attending PR^68,74^) is warranted.

### Conclusions

This qualitative synthesis with application of the health behaviour theory is to our knowledge the first to explore the full range of patient participation behaviour in SM support programmes among patients with COPD, and it has helped explain participation beyond the previously reported socio-demographic and clinical factors. The synthesis helped identify a list of reasons that explained patient participation, and application of theory helped to understand that participation behaviour was influenced by a participant’s attitude and perceived social influences and their perceptions towards the illness and the intervention. As these psychosocial constructs are amenable to change,^18,44^ targeting these key constructs may help improve participation in COPD support programmes and improve health outcomes.

## Figures and Tables

**Table 1:** Characteristics of included studies (*n*=10)

*Study*	*Arnold* *et al*.^34^	*Fischer* *et al*.^35^	*Taylor* *et al*.^38^	*Denn*^36^	*Gysels and Higginson*^37^	*Bulley et al*.^39^	*Keating et al.*^5,11^	*Moore et al*.^40^	*Sohanpal et al.*^10,^^12,^*a*^12^	*Guo and Bruce*^41^
Intervention	PR	PR	PR	PR	PR	PR	PR	PR	SM	PR
Country	UK	The Netherlands	UK	UK	UK	UK	Australia	UK	UK	Taiwan
Aim of the study/research questions	Explore experiences of patients who had been invited to attend PR to gain some insights into the aspects that may influence adherence	Explore factors (role of patients treatment beliefs, goals and practice barriers) predicting dropout in rehabilitation	Explore why participants who took part in a randomised controlled trial of a nurse-led intermediate care package (intervention: 4-week group-based PR programme) declined to take part in the intervention.	Explore patients' understanding and expectations 8 weeks before and after taking part in a PR programme	Understand how people respond to breathlessness: role of PR	How do individuals describe their views about attendance in PR following referral but before attendance?	What are the barriers to uptake of PR for people with COPD? What are the barriers to completion of PR for people with COPD?	Assess the obstacles to participation in PR among COPD patients in a community-based PR programme	Explore reasons for participation to a COPD-specific SM programme from patients with COPD and lay tutors.	Understand the experiences and thoughts attributed to PR attendance and to identify barriers and strategies to establish effective PR
The following was explored from the included studies for the synthesis	Experience of PR adherence	Factors predicting participation and dropout in PR	Reasons for non-participation in the research study of a PR programme	Expectations before and after taking part in a PR programme, which include factors affecting participation	Challenges of participation in PR	Exploration of views about participating in PR, in relation to previous experiences of COPD and its management	Exploration of possible reasons for individual experiences associated with non-attendance and non-completion	Exploring feasibility (the ability or capacity of patients to attend PR) and acceptability (the willingness to attend) among patients who completed or did not complete treatment and patients who were referred but did not complete treatment	Reasons for high and poor attendance in a COPD SM programme	The experiences and thoughts attributed to PR attendance
Was the study part of a mixed-methods study?	No	No	Yes	No	No	Scoping exercise for larger study	No	No	Yes	No
Sample	20	12	39	5	5	10	18	12	16	25
Type of data collection	Individual interviews	Individual interviews	Individual interviews, face to face and by telephone	Focus group	Observations, interviews and field notes	Individual interviews	Individual interviews, face to face and by telephone and observational memos	Interviews (individuals were allowed to have a supporter)	Individual interviews	Focus groups and individual interviews
Primary study underpinned by behavioural theory	No	No	No	No	No	No	No	No	No	No
Emergent themes and subthemes from primary studies on participation and/or completion	(1) Experience of adherence to PR: positive influence of the referring medical practitioner —self-help. (2) Experience of non-adherence to PR: —negative influence of the referring medical practitioner —social support and motivation. (3) Experience of adherence to PR.	(1) Reasons for referral to rehabilitation. (2) Beliefs about PR: —anticipated benefits of participation in the PR programme —concerns about participation in a PR programme —anticipated reasons for dropout.	(1) Travel to and location of pulmonary rehabilitation class {*n*=19/39} (48.7%). (2) Perception of benefit {*n*=19/39} (48.7%). (3) Competing commitments or demands {*n*=15/39} (38.5%). (4) Poor or negative understanding of the research study {*n*=12/39} (30.8%). (5) Past negative experience(s) {*n*=11} (28.2%). (6) Perception of health status {*n*=10/39} (25.6%).	(1) Stoicism (2) Fear (3) Comradeship (94) Empowerment (95) Concept of severity	(1) Pulmonary rehabilitation (2) Challenges (3) Benefits	(1) Desired benefits of attending PR. (2) Evaluating threat of exercise. (3) Attributing value to PR.	Did not attend major themes: (1) Getting there —lack of transport —poor mobility —cost. (2) No perceived benefit —will not make any difference —already exercising enough. (3) Being unwell —COPD —other medical. minor themes: (1) Competing demands (2) Age (3) Fatigue (4) Timing of programme. Did not complete major themes: (1) Being unwell —pain —other medical —exacerbation of COPD. (2) Getting there —lack of transport —poor mobility —cost. Minor themes: (1) Timing of programme (2) Fatigue (3) No perceived benefit (4) Lack of social support (5) Competing demands.	(1) Feasibility —co-morbidities —carer responsibility. (2) Acceptability —perception of PR for COPD —perception of exercise for COPD —presentation of information about PR —unwillingness to prioritise PR —stigma of smoking —suitability of group activity —view of professionals —recommendations of acquaintances —location of PR centre —role of experienced fellow patient —timing.	(1) Reasons for poor attendance —not ill enough —physical/psychological limitations —other obligations. (2) Reasons for high attendance —wanted to learn about SM —socialising —altruism.	(1) Building confidence. (2) Perceiving immediate tangible results. (3) Readiness and Access.
Modified CASP quality checklist score (out of 28)	22	25	24	21	24	27	28	27	27	27

Abbreviations: CASP, critical appraisal skills programme; COPD, chronic obstructive pulmonary disease; PR, pulmonary rehabilitation; SM, self-management.

a The study was included in the review as a grey report (2009). This grey report was published in 2012.

**Table 2:** List of emergent descriptive themes with subthemes illustrated with quotations and references reporting each subtheme

*Theme: reasons for attending with subthemes*	*Participant quotation*	*Theme: reasons for not attending with subthemes*	*Participant quotation*	*Theme: reasons for dropping out with subthemes*	*Participant quotation*
*Studies of PR programmes,* n*=9*					
(1) To help themselves to improve health status^a^^,^^34,35,38,40,41^	“giving me the opportunity to help myself and do something positive instead of just taking this, taking that”^34^	(9) Perceived exercise would worsen health^38,40,41^	*“*…and when I got the letter and it said bring trainers and loose clothing I though oh it must be exercise so that’s really what made me not to go.”^40^	(22) Suffering an acute exacerbation of COPD/other conditions^11,37,41^	“Well when you’ve got osteoarthritis you’ve got bone on bone, and of course it’s painful”^11^
(2) Improvement in health was a priority against prior commitments and demands^a^^,^^35^	*“*Even if you have to give up those things...you have to make choices. Do you want to grab a cup of coffee with someone or do you want to work on your health?”^35^	(10) Perception of health status^b^^,11,34,35,37,38,41^	*“*My breathing on exertion would have to get better”^11^	(23) No change in health status after attending one/few sessions^11,37^	“You get people coming in [PR] just once and they really expect a miracle over night. And because nothing has happened they won’t bother coming back anymore”^37^
(3) Gain control of the condition^a^^,^^36,39^	“I try to keep myself moving, and I’ll not give in…”^39^	(11) Lack of perceived benefit to participation in the programme or research study^b^^11,38,40,41^	*“*I mean the thing is if someone goes to see you on this study thing, yeah? And you sit down there for half an hour and you talk and you tell and there must be something you can say “Well, here we are. Try that. That may help you.”^38^	(24) Social isolation can reduce confidence^c^^,11,34,35^	“being on my own there is no-one to give me a bit of a push or encouragement”^34^
(4) Last chance to cope and remain independent^36,39^	“I hope to be able to walk a bit better, to breathe better…just a wee bit more independence. I know it’s not going to be miracles, but I can only try and see if [PR] helps”^39^	(12) Lack of understanding of research study of the PR programme^38^	‘‘I just thought it was a... oh, what can I say... one of these test programmes, do you know what I mean? Like a guinea pig programme.’’^38^	(25) Intensity of the programme^c^^,11,35,40^	“…even though they’re told what to expect, they don’t know what to expect and if they have trouble like-um walk up stairs—Oh God, I can’t do this, I can’t do that here so what am I going to be doing there?”^40^
(5) Referrals by health-care professionals—with explanation for referral^a^^,34,35,37,39,40^	*“*The lung specialist said: … that he had seen patients who were able to take on some activities after participating in a rehabilitation programme. … Perhaps I’ll be able to walk one block then. And that was the doctor’s aim. They also told me: ‘We can’t cure you. But the intention is to get you some more [lung] volume.”^35^	(13) Negative view of the programme from experience and created by others^b^^,38,39^	“I think you do panic…you think that it’s going to be the last time you’re going to breathe to be honest…Aye, if I was able to breathe better I’d be able to…do more”.^39^ ‘‘See, I’ve got a friend that’s got this... He’s got very bad breathing. And I said to him the other day. And he said ‘‘Ah, it’s a waste of time. I was down there,’’ he said. ‘‘Waste of time,’’ he said ‘‘Then we’s sitting there for half the day talking rubbish.’’ So that didn’t help me. I thought. ‘‘Yeah,’’ he said, ‘‘That’s all they done.’’...‘‘No,’’ he said, ‘‘I ain’t going back there no more.’’^38^	(26) No improvements seen^c^^,35^	“Look, if I didn’t notice any improvement _ I mean, after three weeks you can’t tell- but when I’m halfway through the programme and I can’t feel no difference, I would be wasting my time.”^35^
(6) Previous positive and negative experience of PR—social and emotional support from staff and other participants^a^^,^^35,39^	“See, the good thing is you’re being supervised when you’re busy. And then they suddenly say: ‘You’d better see the speech therapist’. And that’s nice. It’s one integrated system. All these people are watching you”^35^ *“*I thought I was going to die off…but I don’t dwell on it, once it’s past it’s past. It doesn’t make me frightened to go anywhere or do anything you know”.^39^	(14) Prior commitments and competing demands^b^^,11,34,35,38^	“when the dates came through we were going on holiday and that was more important”^34^	(27) Training with other participants^c^^,35^	“Well, some are only there for the fun instead of to get better. That’s not what it’s meant for, of course. It can be a nuisance when they’re chattering for 5 or 10 min, sitting on a fitness machine, while in the mean time you could have used it. But you don’t wanna send them away, of course.”^35^
(7) To socialise^34^	“I don’t have many friends so I did use it as a bit of social time”^34^	(15) Burdensome journey^b^^,11,35,37,38,40^	“I just can’t make it because I have no car and I have to walk all the way down to X Rd; that takes me about half an hour”^11^	(28) Burdensome journey^c^^,11,35^	“Oh, one of the other things I do have a problem with is parking. It’s $X every time you go there. That’s very expensive when you’re on a pension.”^11^
(8) Personal benefits and peer support^d^^,^^34,36,41^	*“*You are with people who understand, because they are walking the same walk”^41^	(16) Negative experience with health-care staff in the location of programme^b^^,38,39^	“I went to [the specialist] and she said, “ Well, you needn’t bother coming back here, there’s nothing we can do for you.”^39^	(29) Feel secure at home^c^^,11,35^	“I feel much more secure at home than anywhere else”^11^
		(17) Location of the programme^b^^,38,41^	Not exemplified with quote	(30) Competing demands^11,40^	“So I started going…the wife’s got Parkinson’s disease and she couldn’t cope on her own…I couldn’t leave her so I stopped going…”^40^
		(18) Seasonal weather^38^	Not exemplified with quote	(31) Timing of programme^11,40^	Not exemplified with quote
		(19) Referrals by health professionals—without explanation^b^^,11,34,35,40^	“this may or may not help you”^34^		
		(20) Too old^11^	Not exemplified with quote		
		(21) Timing of the programme mostly^b^^,11,40^	Not exemplified with quote		
					
*Study of SM programme,* n*=1*^12^					
(32) Altruism	“I tried to help, really. In myself, I’m all right…I said I’d go...because I’m interested…if it can help other people, and I think it probably did…”	(35) Physical limitations	“Well the main reason was I live in this corner…and sometimes if I have a good day I can make it and another time I’m fighting for breath halfway through and then I don’t attempt it...My friend when she comes up to take me, she takes me down in the wheelchair and I get in the car and when we come home she brings me back in the wheelchair”	(37) Psychological limitations	“I didn’t [want] to sit through too much because I tend to get a bit depressed and agitated and what have you…I don’t like getting in with a lot of people…”
(33) Wanted to learn about self-management	“...because I didn’t like the way I was going…I thought well the doctors don’t seem to be doing much although I’ve got the inhalers and I thought I wondered if there was anything I could do differently...”	(36) Prior commitments and demands	“...Wednesday morning is my hair dressing day, the hair dresser comes here (home) and we pay her here, then I get my hair done, I can’t wash it (hair) myself I can’t reach properly with these arms”	(38) Not ill enough	“…basically after 10 to 15 min I realized no I don’t belong here [at the course], ’cos the people there [at the course] are worse than me…these people couldn’t even walk up and down the street without having a...breather so I’m not like that...”
(34) Socialising and wanting to meet others with the same illness	“…from the social side of it as well ’cos when you’re stuck in doors like 24/7 virtually and just to be able to get out and meet other people that are in the same predicament…it’s not so embarrassing…”				

Abbreviations: PR, pulmonary rehabilitation; SM, self-management.

a Includes potential reasons for wanting to attend the PR programme.

b Includes potential reasons for not wanting to attend the PR programme.

c Includes potential reasons that could result in dropping out of the PR programme.

d Reasons for continuing and completing the PR programme.

**Table 3:** Mapping of subthemes onto both behavioural models

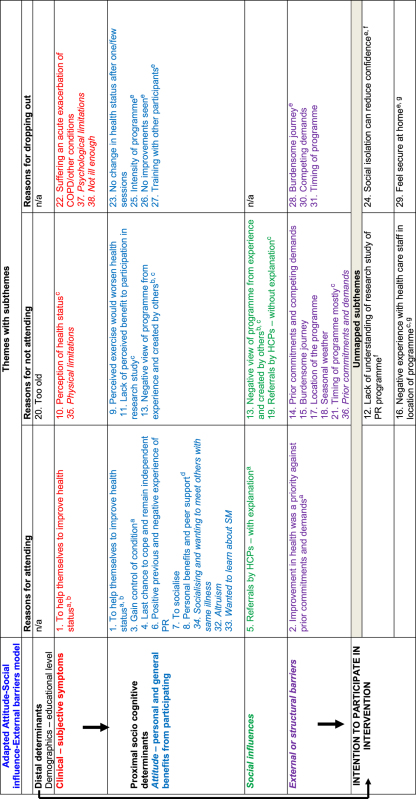
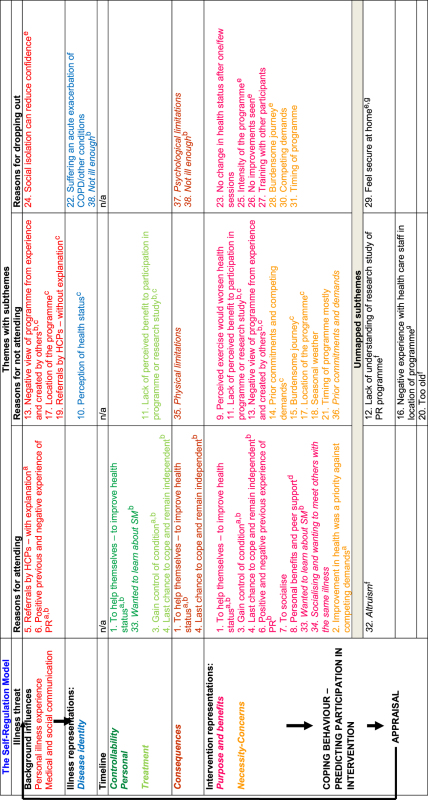
