# Supplementary material for: Understanding patient participation behaviour in studies of COPD support programmes such as pulmonary rehabilitation and self-management: a qualitative synthesis with application of theory
Source: NPJ Prim Care Respir Med. 2015 Sep 17;25:15054–. doi: 10.1038/npjpcrm.2015.54 (PMC4588031; doi:10.1038/npjpcrm.2015.54)
Supplement: Supplementary Appendix 1 [file npjpcrm201554-s1.doc]

| **Appendix 1: Systematic review methodology** |
| --- |
| **The search:**  Search period: 1984 (origin of the first study on chronic disease self-management programme, underpinned by the socio-cognitive theory^78^) to 1 February 2015).  Search strategy: Electronic databases (CENTRAL/CCTR, EPOC, DARE, PubMed/MEDLINE, EMBASE, CINAHL, PEDro, PsychINFO, AMED, BNI, UK and US research registers, ASSIA, ERIC, SSCI); grey reports; and reference lists of relevant studies.  Search terms: (Pulmonary Disease, Chronic Obstructive) OR (Lung Diseases, Obstructive) OR (chronic obstructive adj (pulmonary or lung or airway*) adj (disease* or obstruction or limitation*) OR (Emphysema) OR (Pulmonary Emphysema) OR (Bronchitis, Chronic) OR (Chronic Bronchitis) AND (Pulmonary rehabilitation) AND (Health Education/ or Patient Education as Topic/) OR (Health or patient) adj educat*) OR (Self Care) OR (self adj manage*) OR (psych* or behav*) adj (educat* or manage*) OR (expert adj patient) OR (self help) AND (Patient Participation) OR (patient* or particip*) adj (participat* or attend* or attitude* or motiv* or satisf* or involve* or accept* or refuse* or uptake or recruit* or rate*) OR (Consumer Participation) OR (Patient Acceptance of Health Care) OR (Patient Satisfaction/ or Attitude to Health/ or Aged/) OR Patient Dropouts) OR (drop out* or non attend* or barrier* or non participat*) OR (Program Evaluation) OR (programme evaluation) OR (Self Concept/ or Self Efficacy) OR (self-efficacy) OR (Cognition Disorders/ or Socioeconomic Factors/ or Depression) OR (Predict* adj attend*) OR (Health Services Accessibility) OR (complet* adj rate*).  **Data extraction and quality appraisal**  The data extraction form recorded details on the study characteristics that comprised of: whether the study was part of a mixed-methods study; sample selection; data collection; data analysis; and, results that comprised of *verbatim patient quotes* with authors’ interpretation of the study data and/or authors’ interpretation of the study data not exemplified by *verbatim data.* Patients not referred to PR^40^ and the views of tutors^12^ or health professionals^41^ on the research topic were not recorded and the sample in Table 1 reflects that.  Quality was not an exclusion criteria as even studies of poorer quality may offer new insights, grounded in the data.^81^ Instead, we included all studies to show the comprehensiveness of the literature^82^ on the research topic and the contribution of the included studies towards the synthesis.^83^  The data extraction form with the embedded quality appraisal checklist is available from the authors.  The data extraction, appraisal and synthesis was conducted by RS. The data extraction, appraisal and development of descriptive themes and subthemes was checked for accuracy and discussed with author TM; the ‘mapping’ and generation of analytical themes was checked and discussed with author LS. Author ST was consulted to resolve any unresolved disagreements.  References:  12. Sohanpal R, Seale C, Taylor SJ. Learning to manage COPD: a qualitative study of reasons for attending and not attending a COPD-specific self-management programme. Chron Respir Dis 2012; 9: 163–174.  40. Moore L, Hogg L, White P. Acceptability and feasibility of pulmonary rehabilitation for COPD: a community qualitative study. Prim Care Respir J 2012; 21: 419–424.  41. Guo S-E, Bruce A. Improving understanding of and adherence to pulmonary rehabilitation in patients with COPD: a qualitative inquiry of patient and health professional perspectives. PloS One 2014; 9: e110835.  78. Lorig K, Laurin J, Holman H. Arthritis self-management: a study of the effectiveness of patient education for the elderly. Gerontologist 1984; 24: 455–457.  81. Noyes J, Popay J, Pearson A, Hannes K, Booth A. Chapter 20: qualitative research and cochrane reviews. In: Higgins J, Green S (eds). Cochrane Handbook for Systematic Reviews of Interventions Version 501 [updated September 2008]. The Cochrane Collaboration, 2008.  82. Marston C, King E. Factors that shape young people's sexual behaviour: a systematic review. Lancet 2006; 368: 1581–1586.  83. Dixon-Woods M, Sutton A, Shaw R, Miller T, Smith J, Young B et al. Appraising qualitative research for inclusion in systematic reviews: a quantitative and qualitative comparison of three methods. J Health Serv Res Policy 2007; 12: 42–47. |
