# Supplementary material for: Understanding patient participation behaviour in studies of COPD support programmes such as pulmonary rehabilitation and self-management: a qualitative synthesis with application of theory
Source: NPJ Prim Care Respir Med. 2015 Sep 17;25:15054–. doi: 10.1038/npjpcrm.2015.54 (PMC4588031; doi:10.1038/npjpcrm.2015.54)
Supplement: Supplementary Appendix 2 [file npjpcrm201554-s2.doc]

**Appendix 2: Elaboration of the theoretical models**

**Attitude-Social influence-Self-efficacy model**

De Vries^75^ suggested that the addition of ‘self-efficacy’ from Bandura’s^79^ Socio cognitive theory to ‘attitude’ and ‘subjective norm’ from Fishbein and Ajzen’s^80^ Theory of Reasoned Action was needed to explain behavioural intentions and behaviour. (Constructs ‘Attitude’ and ‘Subjective norm/influence’ has been defined in Figure 1)

***‘Self-efficacy’*** is the personal expectations about the *skills* needed to realise the behaviour. These three variables are proximal social cognitive factors which together are proposed to predict intention to perform the behaviour.

Lemaigre^16^ in their study assessed the above three proximal socio-cognitive constructs, using a structured interview/questionnaire among asthmatics with no prior experience of participation in an asthma SM programme. The questionnaire also included patient characteristics and health outcome measures (referred to as distal factors that can also explain behavioural intention). The findings suggested that significant predictors of intention to attend the self-management programme were higher educational level, perceiving personal benefits (said to reflect a more positive attitude), social influence (defined as higher influence for better self‑care) and self-efficacy (defined as having fewer barriers to participate – having higher self-efficacy expectations). The structured assessment appeared limited in how it defined some terms for example, ‘self‑efficacy’ included only identification of external barriers e.g. no time, living too far away, financial barriers and not internal factors or traditional ratings of confidence. This limitation of not using ‘self‑efficacy’ as a cognitive variable as intended was acknowledged by Lemaigre. Thus, we replaced the ‘self‑efficacy’ construct with ‘external barriers’ and referred to this model as the adapted ‘Attitude-Social influence-External barriers’ model’.

**The Self-regulation model**

Keib^21^ utilising Leventhal’s^76^ ‘Self-regulation model’ and Horne’s^77^ ‘Necessity-Concerns framework’, in their descriptive study demonstrated that an individual with coronary heart disease, their perceptions of the purpose and benefits of cardiac rehabilitation including perceptions of necessity and concerns towards cardiac rehabilitation in relation to their illness representations may influence participation in cardiac rehabilitation.

Reduced participation in cardiac rehabilitation was likely:

if an individual interpreted illness symptoms negatively (disease identity); perceived a coronary heart disease event to be an acute event which can be fixed in short term (timeline); perceived the illness did not affect quality of life (consequence); was unaware or had no knowledge about cause of illness (cause); believed that their own efforts will not help to control disease or the recommended cardiac rehabilitation might not bring benefits (controllability); perceived the exercise component of the programme may be harmful to health (intervention representation); did not perceive participation in cardiac rehabilitation necessary to improve health (perceived necessity); and was concerned about the exercise component of the programme including practical concerns related to attendance (perceived concerns).

References:

16. Lemaigre V, Van den BO, Van HK, De PS, Victoir A, Verleden G. Understanding participation in an asthma self-management program. Chest 2005; 128: 3133–3139.

21. Keib CN, Reynolds NR, Ahijevych KL. Poor use of cardiac rehabilitation among older adults: A self-regulatory model for tailored interventions. Heart Lung 2010; 39: 504–511.

75. de Vries H, Dijkstra M, Kuhlman P. Self-efficacy: the third factor besides attitude and subjective norm as a predictor of behavioural intentions. Health Educ Res 1988; 3: 273–282.

76. Leventhal H, Brissette I, Leventhal EA. The common-sense model of self-regulation of health and illness. In: Cameron LD, Leventhal H (eds). The Selfregulation of Health and Illness Behaviour, 1st edn. Routledge: Oxon, UK, 2003, 42–65.

77. Horne R. Treatment perceptions and self-regulation. In: Cameron LD, Leventhal H (eds). The Self-regulation of Health and Illness Behaviour, 1st edn. Routledge: Oxon, UK, 2003, 138–154.

79. Bandura A. Self-efficacy: toward a unifying theory of behavioral change. Psychol Rev 1977; 84: 191–215.

80. Fishbein M, Ajzen I. Belief, Attitude, Intention, and Behavior: an Introduction to Theory and Research. Addison-Wesley: Reading, MA, USA, 1975.
